# Ecosystem Services in Urban Landscapes: Practical Applications and Governance Implications

**DOI:** 10.1007/s13280-014-0503-1

**Published:** 2014-04-17

**Authors:** Dagmar Haase, Niki Frantzeskaki, Thomas Elmqvist

**Affiliations:** 1 Humboldt Universität zu Berlin, Berlin, Germany; 2 Helmholtz Centre for Environmental Research – UFZ, Leipzig, Germany; 3 Dutch Research Institute for Transitions (DRIFT), Rotterdam, The Netherlands; 4 Stockholm Resilience Centre, Stockholm, Sweden

**Keywords:** Urban ecosystem services, Review, Concepts, Practical applications, Governance

## Abstract

Urban landscapes are the everyday environment for the majority of the global population, and almost 80 % of the Europeans live in urban areas. The continuous growth in the number and size of urban areas along with an increasing demand on resources and energy poses great challenges for ensuring human welfare in cities while preventing an increasing loss of biodiversity. The understanding of how urban ecosystems function, provide goods and services for urban dwellers; and how they change and what allows and limits their performance can add to the understanding of ecosystem change and governance in general in an ever more human-dominated world. This Special Issue aims at bridging the knowledge gap among urbanization, demand creation, and provisioning of ecosystem services in urban regions on the one hand and schemes of urban governance and planning on the other.

## Introduction

Currently, we are entering an urban era (Seto and Reenberg 2014), and 75 % of the world population is projected to live in cities and their peri-urban surroundings in 2050 (UN World Population Prospects 2012). Urban landscapes are therefore becoming the everyday environment for the majority of the global population in the near future. Today, almost 80 % of the Europeans already live in cities and urban areas, and there is no sign that this urban trend will abate (Haase 2014). The continuous growth in the number and size of urban areas along with an increasing demand on resources and energy poses great challenges for ensuring human welfare in cities while preventing an increasing loss of soil, habitats, resources, and biodiversity (Haase et al. 2013). The understanding of how urban ecosystems function, provide goods and services for urban dwellers; and how they change and what allows and limits their performance can add to the understanding of ecosystem change and governance in general in an ever more human-dominated world (Elmqvist et al. 2013).

Urbanization is a complex social, economical, political, and technological process, and there are no uniform patterns of urbanization. Urbanization manifests itself primarily in creating urban landscapes with densification, expansion/sprawling, and shrinkage patterns. The way these patterns emerge and their impact on land and the environment require new methods and new approaches that consider not only the complexity of urbanization dynamics but also interdependencies between drivers, impacts, and responses to these dynamics.

There is a growing research agenda exploring the ecological dimension of urbanization, but this is still young in many regards and yet unbounded by theory or set of frameworks (Cadenasso et al. 2008; Elmqvist et al. 2013). While connections and feedbacks with the hinterland that supported growing urban centers were often apparent in the distant past, this has increasingly been lost in a globalized world (Elmqvist et al. 2013). A current neglect of a social–ecological perspective and a disconnect between the urban and the rural may result in that important feedback mechanisms remain invisible, misinforming policy, and action with large consequences for global sustainability. One aim with this Special Issue is to reintroduce a social–ecological perspective on urban development and contribute to a redefinition of urban sustainability through making invisible feedbacks and connections visible.

Urban ecological systems are deeply situated in the functioning of society, and as such have unique drivers and selection pressures (Elmqvist et al. 2013). A social–technological approach has, until now, been a traditional way of analyzing urban complexity with a focus on how technological innovations drive change in cities and how cities are the living laboratories where technologies are hybridized and diffused (Hodson and Marvin 2010; Frantzeskaki and Loorbach 2010; Geels 2011). The social–ecological systems’ approach to urban ecosystems can offer a new understanding of the synergies, interdependencies and trade-offs between society and ecosystems. It is in cities where a social–ecological co-production of ecosystem services (ES) and society might open new ways for ensuring resilience and livability (Gómez-Baggethun et al. 2013).

In line with this, an urban social–ecological approach (Berkes and Folke 1998) will be increasingly necessary to succeed in enhancing human well-being in urban areas in the face of new and complex challenges such as climate change (Bowler et al. 2010; Ernstson et al. 2010; Chelleri and Olazabal 2012), migration (Seto et al. 2011), shifting and globalized economic investment (Childers et al. 2013), and urban land teleconnections (Seto et al. 2012). Mismatches between spatial and temporal scales of ecological processes and patterns on the one hand, and social scales of use, monitoring and decision-making on the other, have in the past not only limited our understanding of ecological processes in urban landscapes, but have also limited the integration of urban ecological knowledge into urban planning (Kabisch and Haase 2014). Of importance is that the city can serve as laboratory: a space fertile with cultural, social, spatial, temporal, institutional, and biological diversity from which novel ideas can emerge to be tried and tested (Knapp et al. 2008; Nevens et al. 2013). It is here among the bulk of the population, at the point of greatest consumption, that we should be engaging with questions of ecological functionality and environmental sustainability (Grimm et al. 2008).

While the term ‘urban ecology’ was used in sociology and planning schools with variable meaning through the last century (Blanco et al. 2009), urban ecology as a subdiscipline of ecology only emerged in the 1970s in response to a growing awareness of human impact on the natural environment, and the role of cities in this regard (Cadenasso et al. 2008; McPhearson et al. 2013). This legacy has seen both the scientific and planning realms brought together in urban ecology (Pickett et al. 2004; Breuste et al. 2013), which continues to strive to integrate both fundamental and applied research. The dialog between science and policy has revealed that there is a need for better understanding as to what can foster resilience and contribute to livability in urban areas by strengthening/sustaining urban ecosystems. Essentially cities make for heterogeneous landscapes of high temporal and spatial diversities, and urban ecology explores the links and relationships—be they positive or negative—between the ecosystems and species that make up this complex matrix and the associated human activities (Pickett et al. 2004; Kabisch and Haase 2012, 2014).

At the same time, there is a growing research interest in examining what are the factors (contextual, cognitive, demographic or societal) that influence which ES are perceived important or recognized vital by urban dwellers and urban planners therein. We now understand that it is both cultural and biological diversities that underpin resilience and sustainability (Andersson 2006; TEEB 2010). This raises the question of how the social and ecological dimensions and their dynamics can be considered in creating a sound analytical framework that informs planning and governance. To support this, a growing empirical base confirms that urbanization profoundly affects how we connect with and use natural resources. How these impacts play out, in particular with reference to ecosystem functioning and biodiversity, is not yet well understood (Elmqvist et al. 2013; Haase 2012).

Urban ecological and socio-ecological research in Europe, like in other areas of the world, has largely been done by isolated research entities. We have a strong understanding of many aspects of the functioning ecology of the city, but need to start taking a more holistic and integrated approach to our empirical work in the future, in keeping with global trends.

The work presented in this Special Issue *Ecosystem Services in Urban Landscapes: Practical Applications and Governance Implications* should serve as a basis to forward urban social–ecological system approach work in Europe, both with respect to growing the empirical understanding of the ecology in the city, which this issue has shown to be nuanced and relevant, and also toward the less explored ecology of the city as a whole. New conceptual areas worthy of exploration are numerous, but some of the outstanding city-scale questions would relate to the areas of ecosystem service delivery, the role of carbon fluxes, the role of heat, the role of soil functions and a greater understanding of, for example, social connectivity and human health with respect to urban green space.

This Special Issue aims at bridging the knowledge gap among urbanization, demand creation and provisioning of ES in urban regions on the one hand and schemes of urban governance and planning on the other. There is an evident opportunity to generate a new research agenda in a way that would allow for a much more significant cross-discipline and practice engagement. While the geographic focus of this Special Issue is specific, these final points are surely universal. The urban landscape provides a public space for the cross-fertilization of minds and various disciplines, enabling a new perspective on man in nature—one that could place human well-being at the core, break the artificial and largely culturally biased divide between the pristine and the human-dominated ecosystems, and contribute to the creation of a new language, with signs, concepts, words, tools, and institutions that would gather rather than divide, broker conflicts rather than create them, and establish responsible environmental stewardship at the heart of public interest.

## Structure of the Special Issue

### Part I: Setting the Scene

This part introduces new conceptualizations and theoretical contributions for characterizing need and provisioning of ES in urban landscapes and along rural–urban gradients.

Haase et al. (2014) start with a quantitative review about research on urban ecosystem services (UES). The results of the review show in which regions of the world research on UES is carried out. It discusses at which spatial scale this research is done, that is whether local neighborhoods, cities or entire urban regions are considered. Moreover, the authors link the research on UES undertaken so far to important impacts of urban developments such as climate change, the urban heat island, urban footprint etc.

Schewenius et al. (2014) argue that urban features that are resilient and sustainable require an integrated social–ecological approach to urban policymaking, planning, management, and governance. They introduce the Urban Biodiversity and Ecosystem Services (URBES) Project and the Cities Biodiversity Outlook (CBO) Scientific Foundation as new social–ecological contributions to emerging urban resilience and ES research and practice. These two projects represent a growing tool kit useful to local decision-makers and planners for integrating biodiversity and ES in urban development, design and governance mechanisms.

Andersson et al. (2014) study urbanization as a major driver of global environmental change and tend to disconnect urban residents from the biosphere that supports them. Within city, green infrastructure (GI) can offer opportunities and new contexts for people to become stewards of ES. The study analyses cities as social–ecological systems, synthesizes the literature, and provides examples from more than 15 years of research in the Stockholm urban region, Sweden. The social–ecological approach spans from investigating ecosystem properties to the social frameworks and personal values that drive and shape human interactions with nature. The key findings of the empirical work include insights on regulating UES and their stewardship with enabling institutions (e.g., property rights), social networks and involvement of local user groups in green area management and governance. The results highlight the importance and complexity of stewardship of URBES and of the planning and governance of urban GI.

Wurster and Artmann (2014) present a new methodological approach to evaluate UES at a site level. Based on a multi-scale approach, a method for selecting, mapping, and non-monetary assessment of UES of representative sites and its site-specific elements is presented. By using ecosystem service providing and reducing elements as an assessment basis, a concept is developed which allows for the identification of trade-offs and synergies between structures and ES provisioning potential as well as demand and supply of ES. The conceptual design is supplemented by examples of the case study city Salzburg, Austria. The framework enriches the scientific debate about how comparable studies evaluating the provision of ES on a site scale within different cities can be achieved by finding a balance among detail, accuracy, time, and data effort.

### Part II: Providing Ground

This part introduces new interdisciplinary and new integrated analytical tools as practical applications for investigating urban environments, particularly urban green spaces. In their conclusions, these papers also address methods in need to still be developed for examining and exploring UES in cities.

Baró et al. (2014) map regulating ES delivered by urban trees in Barcelona, Spain. The authors argue that an increasing number of scientific studies highlight the importance of ES provided by urban forests to enhance quality of life in cities, yet these services are rarely considered when setting environmental policy targets. The authors apply the Urban Forests Effects (UFORE) model to quantify biophysical and economic values of two ES (air purification and climate regulation) and one ecosystem disservice (air pollution due to biogenic emissions). The results show that the effect of these regulating services is relatively modest compared with to total city levels of air pollution and greenhouse gas emissions. However, our results also show that air purification for particulate matter and climate regulation by urban forests can contribute substantially toward meeting city policy targets of air quality and climate change mitigation.

Voigt et al. (2014) undertook an assessment of urban green spaces in Berlin, and Salzburg showed interest in recreational services provided by urban green spaces. The paper discusses the results of two surveys which include quantitative and qualitative aspects as well as the consideration of future land-use conflicts and the willingness to pay for park conservation. In both cities, open green spaces are appreciated to enrich the everyday life and to be an important factor regarding the quality of life within a city. Even owners of private gardens frequently visit open green spaces, leading to the assumption that private gardens are not able to completely fulfill all recreation desires people have. The differences in the structural equipment are resulting in the attraction of different user groups. In Berlin, the only private park taking entrance fee for the maintenance of the specific beauty and diversity of the site reports a core audience of elderly people compared to the public parks that show a more varying visitor audience. In Salzburg, those visitors are willing to cover longer distances if the park corresponds with their ideas of naturalness. Especially having in mind the trend of ongoing densification and the discussion of the revocation of protection status of urban green, for both cities, these aspects clearly show the necessity and worthiness of protection of urban green spaces along with different designs, adapted to the needs and desires of the people.

Giergiczny and Kronenberg (2014) present a choice experiment to value street trees in the city center of Lodz, Poland, and the broader context of how valuation results helped one to improve the governance of UES in this city. Based on a simplified inventory of trees in the very center of the city, the authors prepared a set of hypothetical programs, assuming changes in the length of three different categories of streets. Different programs put varying emphasis on different ways to increase the numbers of trees, along with different levels of a hypothetical tax that would have to be paid by respondents to implement a given program. The study indicated that the 400 surveyed Lodz residents were willing to pay the highest price for greening those streets where currently there are few or no trees. In general, people were willing to pay for planting trees in the city center. This is an important argument in the public debate not only on the new development strategy for the city but also for the broader context of governing UES in Poland.

### Part III: Steering on the Ground

This part discusses the implications of the new concepts and analytical approaches for urban governance and planning policy at current and in the future in order to achieve more sustainable and resilient cities.

McPhearson et al. (2014) present challenges and opportunities for improving GI and urban biodiversity in New York City. The authors review plans, policies, and organizational efforts to improve GI and biodiversity that affect the provisioning of ES at the city and regional levels. The analysis shows that NYC has made significant progress in improving the environmental quality of its urban ecosystems and in the provisioning of a broad range of UES. Three elements are key to this progress: (1) coherent governmental support in the form of an overarching long-term planning document, PlaNYC and the NYC GI Plan; (2) systematic investment in natural areas, GI and civic engagement by a rich variety of organizations; and (3) a commitment to the acquisition of data that facilitate informed decision-making. In addition to this progress, gaps in governance mechanisms are highlighted for maximizing the potential of biodiversity and GI to meet the growing demand for ES.

Hansen and Pauleit (2014) provide a conceptual framework for multifunctionality in GI planning for urban areas. GI and ES are promoted as concepts that have potential to improve environmental planning in urban areas based on a more holistic understanding of the complex interrelations and dynamics of social–ecological systems. However, the scientific discourses around both concepts still lack application-oriented frameworks that consider such a holistic perspective and are suitable to mainstream GI and ES in planning practice. This literature review explores how multifunctionality as one important principle of GI planning can be operationalized by approaches developed and tested in ES research. Specifically, approaches developed in ES research can help one to assess the integrity of GI networks, balance ES supply and demand, and consider trade-offs. A conceptual framework for the assessment of multifunctionality from a social–ecological perspective is proposed, which can inform the design of planning processes and support stronger exchange between GI and ES research.

Artmann (2014) deals with the management of soil sealing for securing UES in Germany and whether or not it is a question of lack of instruments or political will. Although there is a broad political commitment to stop further sealing, no reversal of trend can be observed in Europe. This paper raises the questions whether a lack of instruments is the reason for further increase, or political–institutional, economical or informational constraints prevent an efficient management and, finally, who has competences to steer soil sealing and who are steering addressees. The analysis is conducted in the growing Munich and the shrinking Leipzig, Germany, analyzing a broad mix of planning-legal, economic-fiscal, cooperative and informational instruments as well as interviewing experts. Results show that the legal basis in Germany is sufficient. But a lack of fiscal instruments, political will and public soil competence promote further sealing.

Frantzeskaki and Tilie (2014) explore whether Rotterdam City has the governance capacity in terms of processes at place, and the attention in terms of vision and strategy to take up an integrated approach toward urban resilience. The authors adopt an interpretative policy analysis approach to assess the dynamics of urban ecosystem governance considering interviews, gray literature and facilitated dialogs with policy practitioners. They show the inner workings of local government across strategic, operational, tactical, and reflective governance processes about the way urban ecosystems are regulated. Despite the existing capacity to steer such processes, a number of underlying challenges exist: need for coordination between planning departments, need to ease the integration of new policy objectives into established adaptive policy cycles and need to assess the lessons learnt from pilots and emerging green initiatives.

## Take Home Messages

Research on UES involves large areas across the globe, but there are still many white spots in Africa, Latin America or Russia poorly investigated, leaving us with an incomplete picture on ES provisioning in cities. But the research presented in this Special Issue also shows the variety of goods and services that are provided in cities and their surroundings to improve human quality of life. What is more, the studies prove that in both quantitative science and urban planning, methods and tools exist to explore UES at different spatial scales to really underpin the concepts and frameworks developed in ES research so far.

## Remaining Challenges

Analyzing how urban ecosystems function, provide goods and services for urban dwellers; and how they change, and what allows and limits their performance can add to the understanding of social–ecological dynamics and suggest new avenues for governing and managing urban system for resilience. It remains for future research to demonstrate how the UES framework can provide bridging pathways and processes toward developing urban resilience plans and policies, governance mechanisms that enable polycentrism and integration, and stewardship strategies to help achieve demand and aspirations for sustainable urban growth, human health and well-being, and paving in this way pathways to urban resilience. Remaining challenges include the following:

- What are the new conceptual notions and frameworks that can advance our understanding of urban resilience using the lens of ES?
- What are the new methods and tools for ES assessment and valuation toward benchmarking? What are the cross-scale and scale-sensitive assessment, mapping and modeling ES tools that could bridge local to global scales?
- What are the lessons learnt from applying ES as tools for resilience assessments and planning?

We invite the research community to explore these possibilities and the implications it may bring for good urban governance from local to global scales. In an urbanizing planet, we believe that a social–ecological approach will center-stage learning and science advancement for urban sustainability and resilience: An approach we expect to develop within the emerging Future Earth initiative.
